# Assessment of Pedestrians’ Head and Lower Limb Injuries in Tram–Pedestrian Collisions

**DOI:** 10.3390/biomimetics9010017

**Published:** 2024-01-01

**Authors:** Yong Peng, Zhengsheng Hu, Zhixiang Liu, Quanwei Che, Gongxun Deng

**Affiliations:** 1 Key Laboratory of Traffic Safety on Track, Ministry of Education, School of Traffic & Transportation Engineering, Central South University, Changsha 410075, China; yong_peng@csu.edu.cn (Y.P.);; 2 CCRC Qingdao Sifang Co., Ltd., Qingdao 266000, China

**Keywords:** tram–pedestrian collisions, head injury, lower limb injury, FE models

## Abstract

Analysis of pedestrians’ head and lower limb injuries at the tissue level is lacking in studies of tram–pedestrian collisions. The purpose of this paper therefore to investigate the impact response process and severity of pedestrians’ injuries in tram–pedestrian collisions, using the Total Human Model for Safety (THUMS) pedestrian human body model together with the tram FE model. Two full-scale tram–pedestrian dummy crash tests were performed to validate the FE model, and the total correlation and analysis (CORA) score of head acceleration yielded values of 0.840 and 0.734, confirming a strong agreement between the FE-simulated head responses and the experimental head kinematics. The effects of different tram speeds and impact angles on pedestrians’ impact response injuries and the differences were further analyzed. The results indicate that direct impact of the lower limb with the tram’s obstacle deflector leads to lower limb bone shaft fractures and knee tissue damage. Neck fling contributed to worsened head injury. Coup contusions were the predominant type of brain contusion, surpassing contrecoup contusions, while diffuse axonal injury was mainly concentrated in the collision-side region of the brain. Pedestrians’ injuries are influenced by tram velocity and impact angle: higher tram velocities increase the risk of lower limb and head injuries. The risk of head injury for pedestrians is higher when the impact angle is negative, while lower limb injuries are more significant when the impact angle is 0°. This study provides practical guidance for enhancing tram safety and protecting pedestrians.

## 1. Introduction

Trams are urban light rail transit vehicles that operate on tracks and share the right of way with other road users, including pedestrians [1]. With their advantages in terms of ecological friendliness, cost economy, scenic routes, and almost no restrictions on installing of stops [2], tram networks have expanded significantly worldwide. As of 2015, over 15,600 km of tram lines had been constructed in 388 cities globally [3]. The growing tram network means that trams interact more frequently with other users, particularly pedestrians who are more vulnerable in traffic situations [4]. According to an analysis of accident statistics, a total of 7535 tram–pedestrian collision crashes occurred in Australia, Sweden, Switzerland, and Germany between 2000 and 2021, resulting in 8802 injuries or fatalities [5]. Based on 259 accident reports between 2000 and 2021 from Swedish emergency hospital departments, injuries to pedestrians in tram collisions were mainly represented by cerebrum injuries, cerebellum injuries, skull fractures, thorax internal organ injuries, rip fractures, pelvic fractures, femur fractures, and tibia fractures [5]. Pedestrians face a higher risk of death when involved in tram collisions compared with automobile collisions at the same speed. Even at low impact speeds of 5–10 km/h, fatalities can occur due to the tram’s front ergonomics, which lead to the full transfer of energy to the weak pedestrian body [6]. Therefore, it is necessary to investigate pedestrians’ injuries in tram–pedestrian collisions.

The areas with the highest risk of tram–pedestrian collisions are public transport spots and pedestrian crossings [6]. Since tram drivers often operate their vehicles at low speeds due to caution, pedestrians may have a false sense of security and be unaware of the potential crash risk, resulting in risky situations [7]. Nevertheless, the rate of most major trauma cases of tram–pedestrian impacts is highest in tram-related crashes [8]. Among tram–pedestrian crashes, head injuries are the most frequent injuries, mostly presenting with brain tissue injuries and skull fractures, etc. [7,8,9,10]. In blunt impact scenarios, the pedestrian’s head is typically subjected to rotational motion and direct contact force. Rotational motion can result in traumatic brain injuries (TBIs), while direct contact force can lead to skull fractures [11]. TBIs can be categorized into focal brain injuries, including cerebrum contusion and hematoma, and diffuse brain injuries (DBIs), including concussion and diffuse axonal injury (DAI) [12,13,14]. Additionally, the lower extremities, which are the main initial point of contact in collisions, often receive severe injuries, mostly in the form of knee dislocations, femur fractures, tibia fractures, etc. [5,8,9].

Up to now, many studies have been conducted on tram–pedestrian collisions. Grzebieta and Rechnitzer [15] conducted a study on the pedestrian dynamics impacted by different classes of tram in Melbourne, Australia, using the dynamics simulation software MADYMO R7.5. They monitored the head injury criterion (HIC) and the viscous injury (VC) of a human dummy model to evaluate injuries. Their research revealed that severe head injuries to pedestrians were caused by the body’s rotation around the bumper, leading to a collision between the head and the front of the tram. Hynčík et al. [16] studied the effect of tram fenders on reducing injury risk to pedestrians, utilizing injury criteria such as HIC, knee bend, and the thorax trauma index (TTI). They discovered that pedestrians’ knee injuries were primarily caused by tram fenders and were challenging to avoid. Chevalier et al. [17] investigated the influence of tram front-end shape on pedestrians’ head injuries, employing a customized parameterized front-end simplification model. The study found that the risk of head injury was more significant compared with other body regions, and a combination of a large windscreen offset and a high windscreen reduced the likelihood of head injuries to pedestrians. Špička and Špirk [18] conducted crash simulations involving three tramway designs and different pedestrians. The study revealed that the pedestrian’s size, the phase of the gait cycle, and the collision position significantly affected both the overall motion and the sustained injuries. In terms of real-world collision tests, Tomsovsky et al. [19,20] validated the usability of the Hybrid Ⅲ dummy for tram–pedestrian crash tests and analyzed the kinematics of tram–pedestrian collisions in the case of a side impact at a speed of 10 km/h. Fanta et al. [21] carried out kinematic and HIC analysis of a pedestrian dummy collision with a KT8D5-type tram at speeds of 10 and 20 km/h. All of the aforementioned studies indicate that pedestrian dynamics are obviously affected by the interaction between the lower extremities and the front end of the tram. Direct collisions between the human head and the tram windshield often result in major traumas [17,22]. However, most of these studies were conducted on traditional older trams. Modern fully low-floor trams, which are longer, heavier, and possess higher kinetic energy [23], are widely used worldwide [24], but the lack of structural design for pedestrian crashes has led to more serious tram–pedestrian crashes [23,25]. Furthermore, the aforementioned studies were limited to overall kinematic and dynamic analysis and did not cover tissue-level injuries.

The mechanisms of automobile–pedestrian collisions are well understood, and the front parameters of vehicles have been optimized for pedestrian protection; for example, structure and stiffness design of the main bumper, secondary bumper, and vehicle hood leading edge to enhance pedestrian safety [26,27,28], and optimized design of the engine hood and windshield for head protection [29,30,31]. However, modern trams are typically constructed with rigid streamlined enclosures made of materials such as aluminum alloy and fiber-reinforced plastic (FRP) [32,33]. These trams lack targeted designs specifically aimed at protecting pedestrians. As a result, pedestrians are more vulnerable to severe impact injuries in a tram–pedestrian collision.

In summary, the characteristics of modern trams determine that pedestrians are susceptible to serious head and lower limb injuries in tram–pedestrian collisions; however, there remains a gap in the research on the tissue-level injuries that occur in these situations, and there is a lack of in-depth analysis of the mechanisms of the injuries and the factors that affect them. To bridge this gap, this paper describes a numerical study on pedestrian head and lower limb injuries caused by direct impact in tram–pedestrian collisions. As described in Section 2, a tram–pedestrian coupled finite element (FE) model was established using LS-DYNA 9.71 R7.0 software, which consisted of the THUMS Version 4 AM50 pedestrian model and the simplified modern hydrogen energy fully low-floor urban tram. The boundary conditions were set based on the crash scenario. The tram–pedestrian coupled FE model was validated by comparing with two full-scale physical tram–dummy crash tests. A parametric analysis was performed considering the effect of tram impact velocity and tram–pedestrian crash angle. In Section 3, the kinematics and injury responses of the pedestrian are assessed according to lower limb and head injury evaluation indexes. Section 4 provides comprehensive discussions of injuries to pedestrians, considering the impact velocity and the impact angle. Finally, in Section 5, the conclusions of this study are summarized.

## 2. Materials and Methods

Compared with expensive physical tests and multi-rigid body (MB) models that can represent only kinematics and dynamics, finite element (FE) models are able to effectively characterize the biological tissue level, which is a gap in the existing research. In this paper, finite element analysis was performed in the LS-DYNA software environment, which is explicit simulation software capable of effectively simulating the response of materials to short periods of severe loading. To evaluate brain injury due to impact in tram–pedestrian collisions, a tram–pedestrian crash coupled numerical model was developed using LS-DYNA software and was validated by a full-scale physical collision test between a tram and a dummy representing a pedestrian.

### 2.1. Pedestrian Model

**Figure 1:**
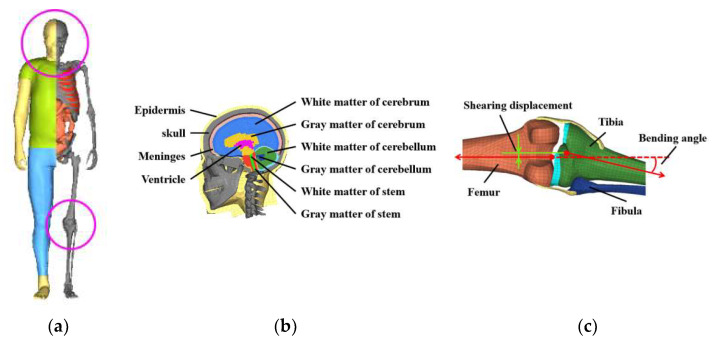
FE pedestrian model: (**a**) Whole-body model; (**b**) head model; (**c**) lower limb model.

In this paper, the THUMS Version 4 AM50 pedestrian model, developed by Toyota Motor Corporation, Aichi Prefecture, Japan and Toyota Central R&D Labs., Inc., Aichi Prefecture, Japan was utilized to simulate the biomechanical responses of pedestrians during impact. The AM50 pedestrian model represents an average-sized adult male with a height of 175 cm and a weight of 77 kg. This model has been verified through a series of impact comparison tests and is widely used in the study of human impact injury biomechanics [34,35,36,37] (Figure 1a). The head of the pedestrian model includes the most essential anatomical features of the human head (the skull, meninges, cerebrum, cerebellum, brain stem, etc.), and the lower limbs include all skeletal components (femur, tibia, fibula, etc.) and major soft tissues (flesh, ligaments, tendons, etc.), as shown in Figure 1b,c. With this detailed representation, the kinematics, dynamics, and tissue injuries of pedestrians in tram–pedestrian collision crashes can be effectively characterized.

### 2.2. Tram Model

**Figure 2:**
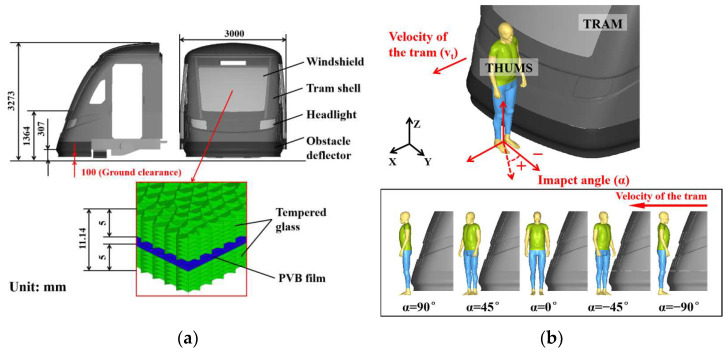
Tram model and collision scenario: (**a**) FE tram model; (**b**) schematic of cases with parameterized factors in different collision scenarios.

Elaborate detailing of the tram’s front-end structure is warranted due to the predominant occurrence of interactions between the vehicle and pedestrians transpiring at its forefront [6,7]. This encompasses a range of components, including the shell, the windshield, the obstacle deflector, etc., which come in diverse materials and shapes. It is noteworthy that components outside this context need to be subject to simplification to enhance computational efficiency. The tram FE model in this study was developed based on the hydrogen energy fully low-floor urban tram, which adopts the general design of a modern tram. The design of the tram follows the main design of the vehicle body structure of the Chengdu Line 18 tram and incorporates a new bogie structure and drive method. The total weight of the tram, without passengers, is 58,000 kg, and it has a ground clearance of 100 mm. The developed tram model consisted of 259,843 nodes and 253,878 elements, including components such as the windshield, aluminum alloy shell, steel frame, headlights, and obstacle deflector (Figure 2). The major metal structures were simplified based on a validated full-scale vehicle FE model [38], while the parts other than the cab were replaced with 1D mass elements. The windshield was modeled using a glass–PVB–glass multi-layer composite structure, which was validated in our previous study [39]. The headlight components were modeled using the same parameters as the glass structure.

### 2.3. Tram-Pedestrian Collision Coupled Model

In previous studies, pedestrians crossing tracks were considered to be vulnerable to the impact of trams and severe injuries [6,7]. This paper is based on a scenario in which a pedestrian crosses a straight railroad track on a clear and dry day, and it is assumed that the driver has already detected the pedestrian before the collision occurs and has taken braking measures as far as possible. The tram–pedestrian collision coupled model was developed, with the tram traveling on a straight and dry urban road, and the pedestrian positioned in front of the tram, as illustrated in Figure 2b. The pedestrian model stood in a default posture on the ground, with the center of mass of the head positioned on the longitudinal profile plane of the tram. The ground was represented using *RIGIDWALL in the LS-DYNA software, and a friction coefficient of 0.7 between the pedestrian and the ground was assigned [37]. The contact between the tram and the pedestrian was modeled using *CONTANT_SURFACE_TO_SURFACE, with the friction coefficient set to 0.35 [17]. The normal walking speed of pedestrians is 1.4 m/s [40]; the pedestrian model speed was set to 1.4 m/s using *INITIAL_VELOCITY. The tram velocity was set to the corresponding value using *INITIAL_VELOCITY to meet the needs of different simulation scenarios. The effect of different gait stances was not considered in this particular study. According to CJT417-2022 [41], the average deceleration during emergency braking for a low-floor tram is 2.25 m/s^2^, which represents the maximum braking force applied by the tram driver. Consequently, the deceleration of the tram FE model was set to 2.25 m/s^2^ using *LOAD_BODY_GENERALIZED to align with this standard.

### 2.4. Validation of the Coupled Model

**Figure 3:**
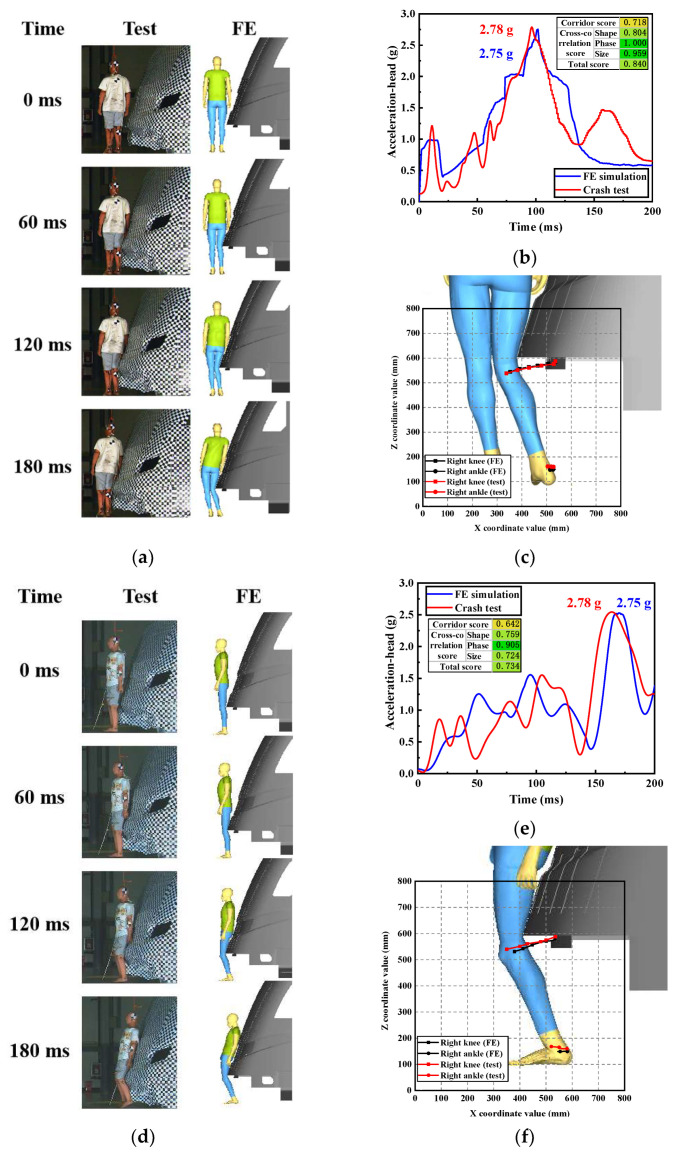
Model validation: (**a**) kinematics comparison of Test 1; (**b**) head acceleration comparison of Test 1; (**c**) comparison of pedestrian’s lower limb behavior histories of Test 1; (**d**) kinematics comparison of Test 2; (**e**) head acceleration comparison of Test 2; (**f**) comparison of pedestrian’s lower limb behavior histories of Test 2.

A real-life case with detailed crash information and medical damage reports that meets the requirements of crash reconstruction cannot be found, and because real-life crash tests are not morally and ethically permissible, this paper verifies the dynamics and kinematics behavior of the developed tram–pedestrian collision model through tram–dummy impact tests. As shown in Figure 3, a Hybrid Ⅲ 50th male pedestrian model provided by HNSAF was used to represent the pedestrian, which has been validated for use in tram–pedestrian crash tests [19,20]. Two real-world full-scale tram–dummy impact tests were conducted in 2023 at CRRC Qingdao Sifang Co., Ltd., Qingdao, China. In both tests, the dummy stood firmly on both feet on the dry concrete ground, with the center of mass of its head positioned on the longitudinal profile plane of the tram. It maintained a natural standing position before the collisions through a rope attached to the head. The other end of the rope was secured to an electromagnetic lock. When the tram made contact with the dummy, the electromagnetic lock released the rope, granting the dummy complete freedom of movement. In Test 1, the dummy was oriented perpendicular to the direction of the Chengdu Line 18 tram’s travel and was impacted at a speed of 4.1 m/s. In Test 2, the dummy was oriented in the same direction as the tram’s travel and was struck by the same tram at a speed of 3.79 m/s. The kinematics were captured using a high-speed camera, while the head acceleration of the dummy was recorded using an accelerometer. Using data obtained from the impact test, the simulation scenario positioned the dummy in an identical standing posture at the same location as in the test setup. The tram speed was set to match that of the tests. It was assumed that the response of the rope fixed to the head had no effect on the dummy, and this was not considered in the finite element model. Additionally, the obstacle deflector was omitted to replicate the shape of the front end of the test tram.

As depicted in Figure 3a, the sequence of events observed in the simulation closely resembled the actual collision scenario in Test 1: the first impact occurred between the knee and the front end of the tram; then, the leg contacted the tram and the torso was leaning towards the tram; after that, the tram collided with the upper limb and the head exhibited a tendency to crash into the windshield. The pedestrian’s head accelerations from the dummy test and the simulation were compared using the correlation and analysis (CORA) objective rating method. This method combines the corridor rating and the cross-correlation rating to evaluate the time-history signals obtained from tests and simulations [42,43]. The result ranges between “0” and “1” depending on the quality of match: a rating of “1” represents a perfect match within defined tolerances and “0” a poor match. As shown in Figure 3b, the total CORA score was 0.840, which proves the good consistency of the head acceleration profile. Figure 3c depicts the trajectories of the pedestrian’s lower limb with respect to the tram datum point. The trajectories of the left knee and left ankle were largely consistent between the test results and the results obtained from the FE model. For Test 2, Figure 3d illustrates the kinematic consistency between the simulation and the collision test: the initial impact was on the back of the knee by the front end of the tram, followed by knee flexion, and subsequently, the pedestrian’s hip and back made contact with the tram. The head acceleration comparison (with a total CORA score of 0.734) in Figure 3e and the comparison of lower limb behavior in Figure 3f both demonstrate strong alignment. By comparing full-body kinematics, head acceleration, and lower limb behavior in both scenarios, the developed model has been validated to accurately replicate the pedestrian–tram collision response.

### 2.5. Simulation Matrix

To investigate the response of pedestrians’ brain biomechanics and lower limbs when subjected to impacts in tram–pedestrian collisions, a parametric study was performed. Two parameters were considered in the collision scenarios: the tram velocity (*v_t_*) and the impact angle (*α*). According to the tramway front end design guide published by STRMTGT Technical Agency for Ropeways and Guided Transport Systems, it is accepted that a pedestrian can survive at an impact speed of no more than 20 km/h [44]. Furthermore, the tram velocity is typically limited to 25 km/h or less at public transport stops and pedestrian crossings [45]. Therefore, as shown in Figure 2b, the tram velocity of the baseline model was set to 20 km/h, the direction of the baseline model was set perpendicular to the running direction of the tram, i.e., 0°. Based on the baseline case, one parameter was varied while keeping the other fixed. For the tram velocity, *v_t_* was set to 12.5–22.5 km/h to obtain 5 cases. Regarding the impact angle, the human model was rotated clockwise and counterclockwise along the vertical axis by ±45° and ±90°, respectively, resulting in 5 cases as shown in Figure 2b. Thus, the simulation matrix was constructed as shown in Table 1, including 9 simulation cases.

### 2.6. Injury Evaluation Index

#### 2.6.1. Lower Limb Evaluation Index

In this study, lower limb long bone fractures and knee soft-tissue injuries were considered to assess the risk of lower limb injuries [46,47,48], which has been effective in previous studies on impact injuries [37]. The average yield stress of lower limb long bones was taken to be 129 MPa (tibia) and 114 MPa (femur), based on cadaver cortical bone tests [49]. These values were used to determine the likelihood of fracture occurrence. The lateral bending angle of the knee and the shearing displacement were calculated to evaluate knee soft-tissue injuries by measuring the vector angle and nodal distance between the two long bone diaphysial axes, as shown in Figure 1c, with respective boundary values of 16° and 14 cm [48].

#### 2.6.2. Head Injury Evaluation Index

The kinematics and injuries of the pedestrian’s brain were studied in this research. The maximum principal strain (MPS) was measured to evaluate the brain contusion, which has been used in previous studies [36,50]. A cut-off value of 0.88 was chosen for MPS, indicating a 50% risk of brain contusion [50]. The cumulative strain damage measure (CSDM) was employed to assess DAI. This involved calculating the cumulative volume fraction of the brain elements with an MPS exceeding the predetermined level [51,52]. In this study, the predefined level was set at 0.15, and there is a 50% risk of DAI when CSDM_0.15_ reaches 55%.

## 3. Results

### 3.1. Kinematics Response in Tram–Pedestrian Collisions

**Figure 4:**
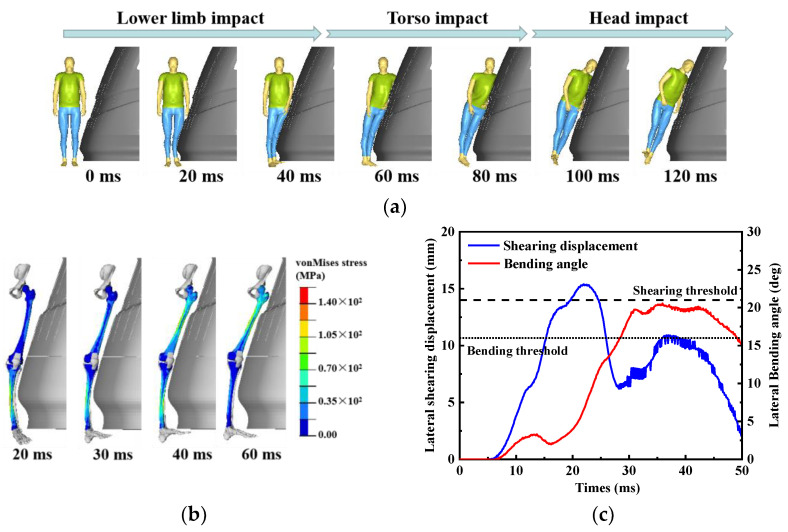

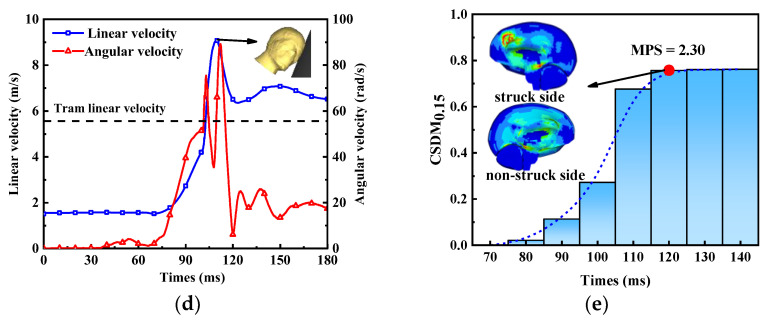
Impact process of the baseline case: (**a**) kinematics process of the whole body; (**b**) stress distributions and kinematics of lower limbs; (**c**) shearing displacement–time curve and bending angle–time curve; (**d**) head linear velocity–time curve and head angular velocity–time curve; (**e**) the CSDM_0.15_–time curve.

The whole-body kinematics process of the baseline case is shown in Figure 4a, illustrating a typical crash scenario: an adult pedestrian was struck by a tram traveling at 20 km/h while crossing the street on foot. The direct impact process can be divided into three stages: the lower limb impact phase, torso impact phase, and head impact phase. In the first phase, the tram’s obstacle deflector initially made contact with the lower leg of the pedestrian, with the impact point at the middle of the tibia. Then, under the influence of inertia, the pedestrian’s knee and thigh sequentially collided with the front end of the tram. In the second phase, the lower limb separated from the contact surface, and the pedestrian’s torso gradually collided with the tram from the hip to the shoulders. In this phase, contact and force transmission predominantly occurred in the pedestrian’s torso. Due to the pedestrian’s inertia, the head remained without any structural contact with the tram, demonstrating a tendency to maintain its motion state. The head lagged behind the torso, and no rotational motion occurred [53]. In the final phase, as the torso continued its forward movement, the impact force was transmitted to the neck, resulting in an increase in shear forces on the upper neck and torque on the atlanto-occipital joint [54,55]. As depicted in Figure 4d, it is evident that the head’s angular velocity rapidly increased at 80 ms, indicating rotational motion of the head. Ultimately, the head impacted the windshield due to the influence of neck fling.

### 3.2. Lower Limb Injury Response

The non-struck leg did not directly collide with the tram, and its lower level of injury was not obvious. Therefore, it was not the focus of the analysis. The kinematics and von Mises stress distribution of the femoral and tibial cortical bones on the struck side are depicted in Figure 4b, while the lateral shearing displacement and the lateral bending angle of the struck knee are shown in Figure 4c. In the collision, the tibia was the first location to experience impact, resulting in bending due to the inertial loading of the mass below the impact point. This bending led to von Mises stress in the struck tibia. The maximum von Mises stress of 135.9 MPa occurred at 20 ms in the tibia shaft, which indicated a risk of tibial fracture. Simultaneously, the knee experienced direct impact, resulting in significant lateral shearing displacement that reached its full maximum and exceeded the shearing threshold. Subsequently, the tram impacted the femur and the femoral von Mises stress was significantly elevated and reached a maximum value of 114.2 MPa at 40 ms, and there was a risk of femur fracture at this moment. The tibia was subjected to a combination of forces, including the non-impact leg’s restraint forces, collision force, upper leg inertial force, and moments generated by the foot’s inertial forces. This resulted in external rotation of the tibia and a substantial loading on the knee. The lateral bending angle exceeded the bending threshold, and the lateral shearing displacement reached a second peak, resulting in severe injury to the knee tissue.

### 3.3. Head Injury Response

After the tram collided with the pedestrian’s shoulder, the head’s linear velocity started to increase due to the inconsistent rotation angular velocities of the pedestrian’s body segments. Strain in the brain tissue began to accumulate from 70 ms under the effect of inertia loading (Figure 4e). At 100 ms after the initial collision, the pedestrian’s head collided with the windshield at the bone gap between the parietal and temporal bones (Figure 4d). As a result of the head–windshield impact, the head’s angular velocity decreased, but its linear velocity sharply increased at 95 ms under the effect of the law of conservation of momentum, as the mass of the pedestrian is much smaller than that of the tram. The head’s linear velocity then decreased while rolling sideways, influenced by the coupling effect of pedestrian’s initial velocity perpendicular to the collision force direction, neck restraint, and head–windshield friction. In summary, the head’s linear velocity showed an initial tendency to increase and then decrease, while the head’s angular velocity showed a sharp oscillation. Stress waves were generated at the collision location and propagated within the skull and brain, resulting in positive pressure in the coup brain region and negative pressure on the contrecoup brain region. This process led to rapid accumulation of brain tissue strain, primarily within the brain (Figure 4e). MPS reached a value of 2.30 at 120 ms and CSDM_0.15_ was 76.3%, both exceeding their corresponding cut-off values, which indicated a risk of brain contusion and DAI.

### 3.4. Relationship between Input Parameter and Injury Index

**Figure 5:**
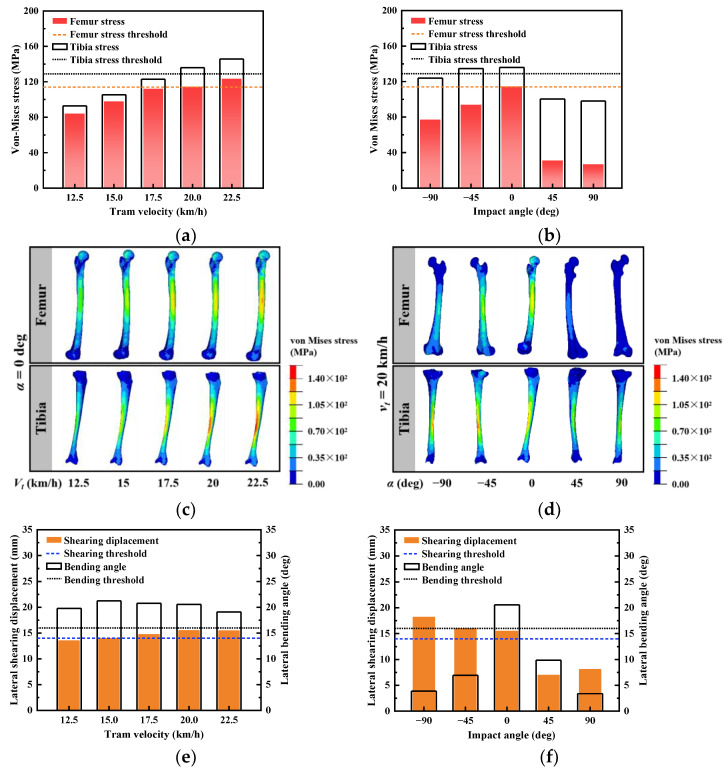
Relationship between input parameter and lower limb injury index: (**a**) maximum von Mises stress in the femur and the tibia for different tram velocities; (**b**) maximum von Mises stress in the femur and the tibia for different impact angles; (**c**) the stress distribution at the maximum value moment for different tram velocities; (**d**) the stress distribution at the maximum value moment for different impact angles; (**e**) maximum knee lateral shearing displacement and lateral bending angle for different tram velocities; (**f**) maximum knee lateral shearing displacement and lateral bending angle for different impact angles.

Figure 5 shows the lower limb injury indexes and stress distribution in struck femurs and struck tibias for different tram velocities and impact angles. Specifically, when the impact angles were ±90 degrees, the pedestrian’s lower limb response exhibited bilateral symmetry, and the left lower limb was selected for analysis.

Regarding lower limb long bone injury indexes, the maximum von Mises stress in the struck tibia and femur was positively correlated with tram velocity and followed an increasing then decreasing trend with increasing impact angle (Figure 5a,b). The maximum stress was concentrated on the shaft of the long bones (Figure 5b,c), with the tibia having a higher risk of bone fracture compared with the femur. For knee tissue injury indexes, the knee’s lateral shearing displacement was positively correlated with tram velocity and negatively correlated with impact angle. However, the knee’s lateral bending angle was not significantly affected by tram velocity but showed a positive correlation with the absolute value of the impact angle (Figure 5c,d). Overall, the knee tissue injury was the most severe in high-speed collision scenarios where pedestrians faced trams.

**Figure 6:**
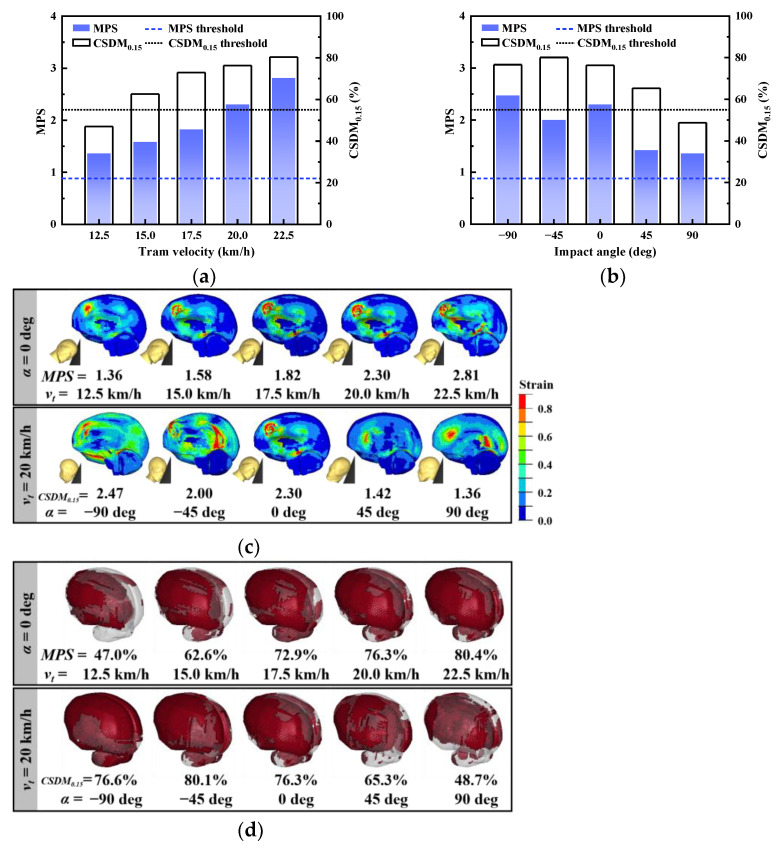
Relationship between input parameter and head index: (**a**) head injury indexes for different tram velocities; (**b**) head injury indexes for different impact angles; (**c**) strain distribution at the moment of maximum MPS; (**d**) the distribution of brain elements exceeding the predetermined MPS level.

Figure 5a,b depict the MPS and CSDM_0.15_ for various tram velocities and impact angles. Figure 6b illustrates the strain distribution of brain tissue at the moment of maximum MPS and the corresponding head collision posture. Figure 6d displays the distribution of brain elements exceeding the predetermined MPS level for different input parameters. Regarding head injury indexes, both MPS and CSDM_0.15_ demonstrated a positive correlation with tram velocity and a negative correlation with impact angle. In all calculated cases, the risk of brain contusion and DAI exceeded the predefined threshold, except for the case where *v_t_* = 12.5 km/h and *α* = 90 deg, where the CSDM_0.15_ was below the predetermined level.

## 4. Discussion

### 4.1. Bone Fracture Severity

According to the reports from Swedish emergency hospital departments, femur and tibia fractures were the main form of lower limb injury for pedestrians in tram crashes [5], which is consistent with the findings of this paper. Additionally, Christine et al.’s study [17] identified instances where tibial accelerations surpassed safety thresholds, consistently exceeding femoral accelerations overall. This suggests a higher risk of tibial fractures compared with femoral fractures, aligning with the findings of this study. However, they did not provide a comprehensive explanation for this conclusion. Therefore, this paper aims to offer additional detailed information at the tissue level and endeavors to elucidate this observed disparity.

The risk of tibial bone fractures in the struck leg increased with higher tram velocities due to the greater impact velocity and energy involved (Figure 5a). This process is similar to the “contact effect of the impactor” observed in car–pedestrian collisions [56], although the point of impact is closer to the distal tibia. The maximum von Mises stress in the tibia consistently increased and surpassed the tibia stress threshold when *v_t_* ≥ 20 km/h. The maximum stress was located in the middle of the tibial shaft and decreased towards the ends (Figure 5b). As the vehicle speed increased, the femoral maximum stress also increased, surpassing the femur stress threshold when *v_t_* = 20 km/h (Figure 5c). The maximum stress in the femur occurred at the point of contact between the thigh and the edge of the prong below the headlight. Therefore, this value was positively correlated with tram velocity and aligned with the risk of tibia fracture. Although the risk of femur fracture aligns with that of the tibia at different tram velocities, it is crucial to consider them separately in the design of pedestrian protection due to the occurrence of two successive impacts at different locations, involving the thigh and lower leg.

**Figure 7:**
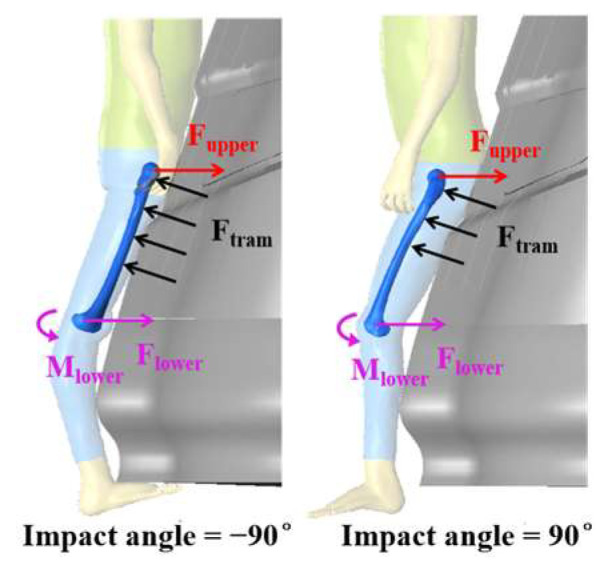
Femur loading in impact angle = ±90°.

When considering the impact angle, pedestrians facing the tram (*α* < 0°) had a higher risk of fracture compared with those facing away from the tram (*α* > 0°), as shown in Figure 5b,d. In the former cases, the collision occurred at the anterior tibial edge, with only a small amount of soft tissue such as the tibialis anterior acting as a cushion. However, in the latter cases, the collision location involved the thick gastrocnemius muscle, and the rotation of the knee joint reduced tibial flexion, resulting in reduced tibial stress. Additionally, the relative collision speed was higher in the case of facing the tram. The loading on the femur is shown in Figure 7, where F_upper_ represents the inertia force from the upper body, F_tram_ is the impact force from the tram, F_lower_ is the inertia force from the lower leg, and M_lower_ is the bending moment from the knee. When the impact angle was 90 degrees, the flexion of the knee reduced the bending moment from the knee, consequently reducing the peak von Mises stress in the femur shaft [37]. Furthermore, the knee bending positioned the collision point closer to the hip, which has abundant fat, providing additional cushioning. These factors made the peak femoral stresses in cases where *α* > 0 much less than in cases where *α* < 0. Specially, in the case of *α* = −90°, each leg shared half of the impact force, so that the unilateral tibial and femoral stresses were less than in the case of *α* = −45°.

### 4.2. Knee Injury Severity

In terms of different tram velocities, the excessive lateral knee bending angle was identified as the main cause of knee tissue injury, and this parameter was not significantly influenced by the tram speed. This is because the lateral bending of the knee was primarily affected by the tram’s frontal profile and was generated by the inertial forces of the leg. In the study by Hynčík [16], a similar phenomenon was observed, where the author suggested that the anterior convexity of the fender led to knee injuries. However, in their study, only the pedestrian’s knee bend was quantified as this metric to assess the phenomenon. In order to comprehensively evaluate knee injury, we quantified and analyzed the shear displacement of the knee. Based on our research, we observed that the knee’s lateral shearing displacement exhibited bimodal characteristics, but the larger first peak, caused by the impact force of the tram, led to knee tissue injury. As a result, the knee’s lateral shearing displacement increased with increasing tram velocity. When *v_t_* ≥ 17.5 km/h, the value of the shearing displacement exceeded its threshold. Therefore, the traveling speed of trams should be strictly limited to no more than 17.5 km/h at stations and intersections where there is a high pedestrian presence, to ensure pedestrian safety. In car–pedestrian crash studies, a low and flat lower bumper design has commonly been employed to limit the rotation of the lower leg and protect the lower limb [57]. Trams could consider incorporating similar bumper designs or decreasing the stiffness of the obstacle deflector to enhance safety.

The impact angle was found to have a significant influence on both the shearing displacement and the lateral bending angle of the knee. The knee’s lateral bending angle increased as the impact angle decreased. In the case where a pedestrian faced a tram during the collision, the patella directly collided with the tram, resulting in a greater horizontal displacement of the proximal tibia. Meanwhile, the latter decreased as the absolute value of the impact angle increased. This is because the longitudinal component of the knee loading caused longitudinal flexion of the knee joint, thereby reducing the damage to the knee tissue from the lateral bending angle. In conclusion, when a tram–pedestrian collision is unavoidable, efforts should be made to maintain a positive impact angle, as it can help reduce knee tissue injury.

### 4.3. Brain Contusion Severity

The collision velocity, which represents the kinetic energy of impact and the contact intensity, showed a significant positive correlation with brain contusion. As the tram velocity increased, the MPS increased monotonically (Figure 6a). Under the condition where the pedestrian velocity was perpendicular to the direction of tram operation, the effect of tram velocity on the location of the high-strain region in the head was not significant. The coup contusion of the pedestrian primarily occurred at the frontal lobe of the brain corresponding to the impact point, while the contrecoup contusion occurred at the midbrain opposite to the impact point.

As the impact angle increased, brain contusion showed an overall decreasing trend. The brain contusion area was more influenced by the impact angle, but the frontal lobe remained the main area where brain contusion occurred. In particular, in the collision scenario with *α* = −90°, there was not only the highest risk of brain contusion but also a high risk of facial bone fracture (including nasal and maxillary fractures) due to the direct action of the impact force on the face. This is also reflected in accident statistics, which show that 57 out of 554 tram–pedestrian collisions in Sweden between 2000 and 2021 involved facial fractures [5].

**Figure 8:**
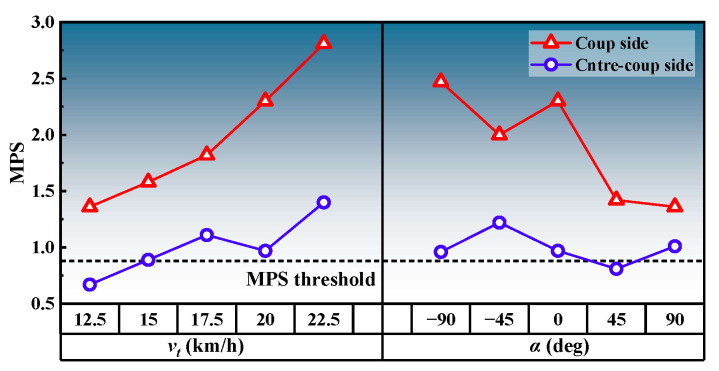
Comparison between coup contusion and contrecoup contusion.

When the pedestrian’s head collided with windshield, a stress compression wave was generated at the collision location. This compression wave propagated to the opposite side of the collision location and formed a tensile wave after reflection [36]. These processes led to coup contusion from compressive strain and contrecoup contusion from tensile strain. Brain contusion on impact is believed to be associated with high linear acceleration [58]. Figure 8 shows a comparison of the MPS on the coup side and contrecoup side, revealing that the coup side had larger MPS than the contrecoup side, indicating more severe coup contusion than contrecoup contusion. This finding is consistent with the contusion distribution observed in rat brain impact tests conducted at the same speed level [59]. This phenomenon is also demonstrated in the accident data: on analyzing 18 tram–pedestrian collision crash cases with multidetector computed tomography (MDCT), 5 of them showed cerebral hemorrhage and edema or subdural hematoma on both the left and right sides of the pedestrian’s head [60]. We speculate that the stress waves at the impact level in this study were small and dissipated during propagation to the opposite side, resulting in the observed brain contusion distribution. Therefore, coup contusion is the primary cause of brain contusion in tram–pedestrian collisions and requires more attention in post-accident diagnosis and treatment.

### 4.4. DAI Severity

As shown in Figure 6b,d, CSDM_0.15_ was positively correlated with tram velocity, and the cumulative strain elements were concentrated on the coup side of the brain. When the tram velocity exceeded 12.5 km/h, CSDM_0.15_ exceeded 55%, implying a DAI risk of more than 50%.

The impact angle affected the CSDM_0.15_ and the area of strain concentration in the brain. The greatest risk of DAI (CSDM_0.15_ = 80.1%) occurred at different impact angles when *α* = −45°, and the strain elements were distributed in the brain, cerebellum, and brainstem. As the impact angle increased, the strain concentration area gradually shifted from the anterior to the posterior part of the head, while the distribution of strain elements in the cerebellum and brainstem gradually decreased.

**Figure 9:**
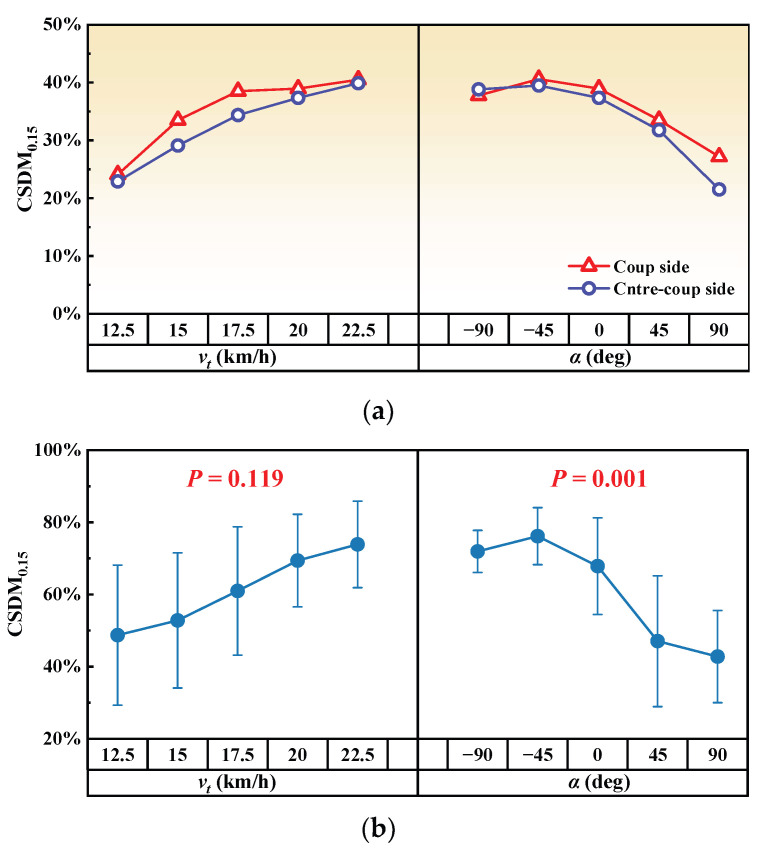
DAI severity analysis: (**a**) comparison between coup side CSDM_0.15_ and contrecoup side CSDM_0.15_; (**b**) results of ANOVA.

In contrast to the MPS observed on both the coup and contrecoup sides, the coup side CSDM_0.15_ was overall slightly larger than the contrecoup side CSDM_0.15_; the difference between the two was not significant, as shown in Figure 9a. This suggests that DAI is uniformly distributed on both sides of the brain in tram–pedestrian collisions, and that careful attention needs to be paid to the mechanical disruption of axons in the cerebral hemispheres and subcortical white matter on both sides of the brain in subsequent diagnosis and treatment. To further analyze the influences of tram velocity and impact angle on CSDM_0.15_, we introduced additional simulation cases and conducted a one-way analysis of variance (ANOVA). For each train speed, 5 collision angle cases were calculated, resulting in a total of 25 cases. As illustrated in Figure 9b, impact angle exhibits significant correlation with CSDM_0.15_ (*p* < 0.05). In general, the CSDM_0.15_ decreased with the increase of impact angle. Meanwhile, the influence of tram velocity was insignificant (*p* > 0.05) within the tram velocity range considered in this paper. F = 2.099 indicated that the mean squared between (MSB) and mean squared error (MSE) were close, implying that the variance of CSDM_0.15_ at different tram speeds was relatively large, while the means were similar, leading to the observed lack of significance. The aforementioned research demonstrates that, in terms of reducing DAI, it is more effective to warn pedestrians to move away from the tram rather than relying on emergency braking.

## 5. Limitation

The primary focus of this study was to analyze pedestrians’ injury patterns in tram–pedestrian collisions, enabling timely and effective radiological and clinical assessments, as well as assisting in injury prevention. The effect of vehicle shape on damage patterns has only been analyzed preliminarily, a shortcoming that we hope will be effectively supplemented in subsequent studies.

Another limitation of this study is the absence of a muscle controller in the employed THUMS Version 4 AM50 pedestrian model. The research findings overlook the impact of muscle tone conditions, potentially leading to disparities in the pedestrian’s kinematic responses compared with real-world accidents. Furthermore, this study employed a digital model with fixed parameters, introducing differences compared with real-world crashes, including variations in collision modes and types of victims involved. In future research, leveraging the THUMS family for collision studies within a more diverse population could contribute to addressing these limitations.

It is important to note that, for safety considerations, the validation experiments in this study were conducted at lower collision speeds. High-speed tests using full-sized trams carried the potential risk of the human dummy falling onto the tracks and possibly causing tram derailment. In contrast, at lower test speeds, the human dummy still effectively represented the dynamic and kinematic responses, enabling the validation of parameters such as friction coefficients, material properties, and modeling method.

## 6. Conclusions

This study investigated the impact response process and severity of pedestrians’ injuries in tram–pedestrian collisions.

1. The results indicate that the direct impact between the lower limb and the tram’s obstacle deflector leads to tibial shaft fractures, as well as lateral shearing displacement and bending of the knee, resulting in knee tissue damage. Tibia fractures and knee injuries are highly sensitive to the velocity and impact angle of the tram.
2. The neck flinging contributes to worsened head injuries in tram–pedestrian collisions. Coup contusion is the primary form of brain contusion, but contrecoup contusion should not be overlooked. DAI is uniformly distributed on both sides of the brain. Compared with tram velocity, the impact angle has a significant effect on DAI.
3. With trams becoming an important component of urban transportation systems, the incidence of tram–pedestrian collisions has increased. This research provides valuable insights for the development of driving regulations in high-risk collision areas, pedestrian protection studies, and the diagnosis and treatment of injuries to pedestrians.

## Figures and Tables

**Table 1:** Parameter variations in the simulation matrix.

Input Parameter	Simulation Matrix					
	Baseline Case	Value Sample (Baseline Case Shown in Brackets)				
Tram speed $v_{t}$ (km/h)	20	12.5	15	17.5	(20)	22.5
Impact angle α (°)	0	−90	−45	(0)	45	90

## Data Availability

The data generated and/or analyzed as well as the source code used in the current study are not publicly available due to their use in an ongoing project, but may be available from the corresponding author on reasonable request.
